# Uniform Selection as a Primary Force Reducing Population Genetic Differentiation of Cavitation Resistance across a Species Range

**DOI:** 10.1371/journal.pone.0023476

**Published:** 2011-08-12

**Authors:** Jean-Baptiste Lamy, Laurent Bouffier, Régis Burlett, Christophe Plomion, Hervé Cochard, Sylvain Delzon

**Affiliations:** 1 INRA, UMR 1202 BIOGECO, F-33610 Cestas, France; 2 Université de Bordeaux, UMR 1202 BIOGECO, F-33610 Cestas, France; 3 INRA, UMR 547 PIAF, University of Blaise Pascal, F-63100 Clermont-Ferrand, France; 4 FCBA, Station Sud-Ouest, Domaine de Sivaillan, F-33480 Moulis en Médoc, France; University of Umeå, Sweden

## Abstract

**Background:**

Cavitation resistance to water stress-induced embolism determines plant survival during drought. This adaptive trait has been described as highly variable in a wide range of tree species, but little is known about the extent of genetic and phenotypic variability within species. This information is essential to our understanding of the evolutionary forces that have shaped this trait, and for evaluation of its inclusion in breeding programs.

**Methodology:**

We assessed cavitation resistance (*P*_50_), growth and carbon isotope composition in six *Pinus pinaster* populations in a provenance and progeny trial. We estimated the heritability of cavitation resistance and compared the distribution of neutral markers (*F*_ST_) and quantitative genetic differentiation (*Q*_ST_), for retrospective identification of the evolutionary forces acting on these traits.

**Results/Discussion:**

In contrast to growth and carbon isotope composition, no population differentiation was found for cavitation resistance. Heritability was higher than for the other traits, with a low additive genetic variance (h^2^_ns_ = 0.43±0.18, CV_A_ = 4.4%). *Q*_ST_ was significantly lower than *F*_ST_, indicating uniform selection for *P*_50_, rather than genetic drift. Putative mechanisms underlying *Q_ST_<F_ST_* are discussed.

## Introduction

The climatic niches of forest tree species are moving faster than the maximum rate of migration, measured by palynology or gene flow analysis, as a direct consequence of the current increase in temperatures due to global warming [1], [2]. Forest tree populations are thus facing new selection pressures and are unable to track their bioclimatic envelope [3] over the time scale at which these changes are occurring. The local adaptation and survival of tree populations in a rapidly changing environment with warmer temperatures and more frequent water shortage is a major concern in efforts to ensure the sustainability of forest ecosystem services. In addition to this trend, climate experts are predicting more extreme climatic events, such as periods of severe drought [4], which will increase mortality rates [5], [6], [7]. These effects on tree mortality highlight the way in which the impact of climate change may depend on the changes associated with extreme events rather than trends [8]. In this context, there is a need to investigate relevant drought tolerance-related traits, to quantify both genetic variation and phenotypic plasticity, which together define the capacity of tree populations to adapt.

From a physiological point of view cavitation resistance is an important trait to estimate drought tolerance. Indeed, dysfunctions of the vascular system of the tree, such as xylem embolism due to cavitation events, is likely to be a key factor governing the mortality of these long-lived organisms [9].When a cavity is formed in the xylem sap under tension (negative pressure), it may spread in the vascular system through intervessel or intertracheid pits, thus compromising the capacity of the plant to transport water [10].

A direct causal link between survival (fitness) and cavitation resistance during extreme drought has been highlighted, based on two lines of evidence suggesting that cavitation resistance is an important adaptive trait. Firstly, assessments of the correlation between cavitation resistance and lethal water potential [11], [12] demonstrated a highly significant linear relationship (r^2^ = 0.9) between these two traits. Secondly, global surveys of cavitation resistance in woody species showed that xeric species are more resistant to embolism than hydric species [13], [14], [15]. These interspecific studies, with adaptive inferences concerning cavitation resistance being rendered robust by the incorporation of phylogenetic information [15], [16], [17], concluded that cavitation resistance-related traits are under natural selection [15], [18]. To validate this evolutionary pattern, a population-level perspective is appropriate, because variation observed across species cannot be assumed to reflect patterns within species.

At the intraspecific level, cavitation resistance can be analyzed by provenance or progeny trials. The few studies carried out to date (reviewed in Table S1) have included only small numbers of individuals (<9) and populations (<5), and it has therefore not been possible to estimate environmental and genetic effects on phenotypic variation accurately. We therefore still know little about the genetic determinism and micro-evolutionary pattern of this hydraulic trait, but such information is absolutely necessary if this trait is to be incorporated into breeding programs and for a more fundamental understanding of the evolutionary basis of tolerance to severe drought.

The aim of this study was to provide the first estimates of heritability, additive variance and population differentiation for cavitation resistance-related traits. We carried out a case study of maritime pine (*Pinus pinaster* Ait.), a species with a fragmented distribution in the western part of the Mediterranean region. The scattered distribution of this species may have prevented or limited gene flow between different groups of populations, promoting high levels of genetic divergence between ecotypes due to genetic drift [19] and/or natural selection (Quezel and Barbero 1998 in [20]). Here, we took advantage of a new technology (high-throughput phenotyping platform for cavitation resistance) to screen for the first time a large number of genotypes from six ecotypes to test the hypothesis that *Pinus pinaster* populations have been subjected to diversifying selection for cavitation resistance. More specifically, this experiment aimed to address the following questions: what is the level intraspecific variation and heritability for cavitation resistance? Can we separate the relative roles of drift and selection in population differentiation for this trait?

## Methods

### Provenance trial and climatic data

We carried out a provenance-progeny trial, in which young trees (six-year-old plants) were planted in December 2003 at the INRA forestry station in the Aquitaine Region (44°44’N, 00°46’W). The mean annual temperature at this site is 13.2°C and mean annual rainfall is 836 mm (1984–2006). The soil is a sandy podzol with a water table rising to about 0.5 m below the surface in winter and descending to a depth of 2 m in late summer. Seedlings were grown in the nursery from open-pollinated seeds collected from 24 natural populations (or ecotypes) in France, Spain, Morocco and Tunisia, to cover the fragmented distribution of *Pinus pinaster*. Each population was represented by 20 to 30 half-sib families. The trial was arranged in a randomized block design (15 blocks) with single-tree plots. Each block contained at least one tree from each half-sib family. There were 600 seedlings per block, giving 9,000 seedlings for the entire experiment.

### Choice of populations

**Figure 1:**
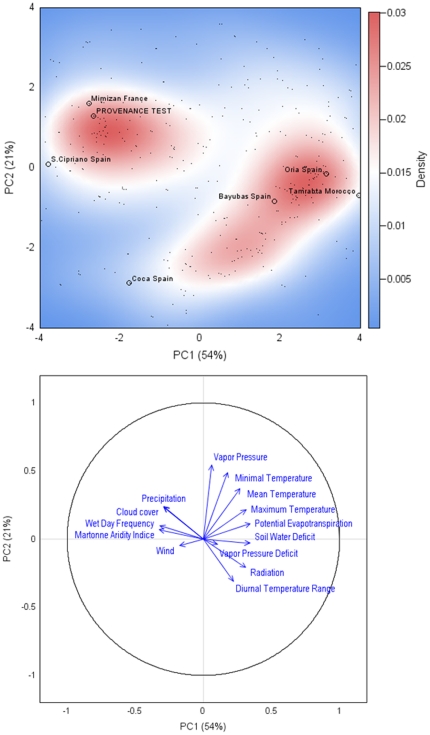
Principal component analysis (PCA) on the [763 population locations x 14 climatic variables] data matrix. Upper panel: The contour plot represents the presence's probability (kernel density estimate) of *Pinus pinaster* population (small black dot) within the bioclimatic envelope representing by PC1 and PC2 axes, accounted for 54% and 21% of the variance, respectively. The studied populations and provenance test are indicated by black circles. PCA was performed with the variables indicated in the methods section. See Table S2, for additional information about the relative contribution of climatic variables to the axes. Lower panel: projection of 14 climatic variables on the subspace spanned by the first two eigenvectors (correlation circle).

The assessment of cavitation resistance is a intensively time-consuming process [21]. We therefore designed a procedure for the selection of a subset of populations representing all the variability of climatic envelope of maritime pine. For a total of 754 grid points covering the entire natural range of the species [22], we first extracted climatic data from the CRU CL 2.0 10′ global dataset for the period 1961–1990 [23], [24], [25]. These data included monthly average precipitation, mean, minimum and maximum temperature, diurnal temperature range, water vapor pressure, cloud cover, wet day frequency, ground frost frequency, radiation, wind, Martonne index, Turc's potential evapotranspiration and soil water deficit. We also derived the vapor pressure deficit from these parameters. Principal component analysis (PCA) was then used to reduce the number of dimensions for the whole set of climate variables (Figure 1). The data were centered and scaled before PCA. The 14 populations were finally placed on the main plane of the PCA (accounting for 76% of the variation, Table S2) and six of these populations (Table 1) were selected as a representative subset of the climatic envelope covered by *Pinus pinaster* species. In each population, eight families (5 half-sibs/family/block) were randomly selected for further analysis.

**Table 1:** Climatic data, location and elevation of the studied maritime pine populations.

Sampling location	Longitude(°)	Latitude(°)	*n*	Elevation(m)	*P*_i_(mm)	*T*_m_(°C)	*VPD_max_*(hPa)	*ETP*( mm)
Bayubas de Abajo (Central Spain)	−2.87	41.52	39	955	561	10.5	11.42	882.9
Coca (Central Spain)	−4.08	41.37	40	788	452	11.9	14.23	718.7
Mimizan (South-western France)	−1.30	44.13	40	37	1176	13.2	7.26	751.59
Oria (South-eastern Spain)	−2.62	37.87	40	1232	451	13.4	14.29	922.59
San Cipriano (Northern Spain)	−8.70	42.13	40	310	1625	13.8	8.54	721.91
Tamrabta (Southern Morocco)	−5.02	33.66	40	1760	550	15.1	18.56	976.54

*n*, number of sampled individuals for hydraulic measurements; *P*_i_ , mean annual precipitation; *T*_m_, mean annual air temperature; *VPD_max_*, maximal of water vapor pressure deficit (in July for all the provenance); *ETP*, annual sum of potential evapotranspiration.

### Sample preparation for the assessment of cavitation resistance

We collected branches, according to the sampling procedure described below, during winter 2009, before 10 am, to avoid native embolism. Needle water potential was lower than -1 MPa, far from the minimum needle water potential in summer (−2 MPa) of *Pinus pinaster* [26]. The branch sample corresponded to the 2007 and 2008 growth units on the 2007 whorl when possible, in order to measure on the same number of rings in each sample. Sampled branches were fully exposed to the sun, longer than 40 cm and with a diameter of 0.3 to 1 cm (<4 years of age). The current needles were removed and the branches were wrapped in wet paper towels and bagged upon collection to prevent dehydration. In the lab, samples were cut under water just before measurement to obtain 0.28 m segments (*i.e.*, much longer than the longest tracheid). Bark was removed from all segments before measurements.

### Assessment of cavitation resistance

**Figure 2:**
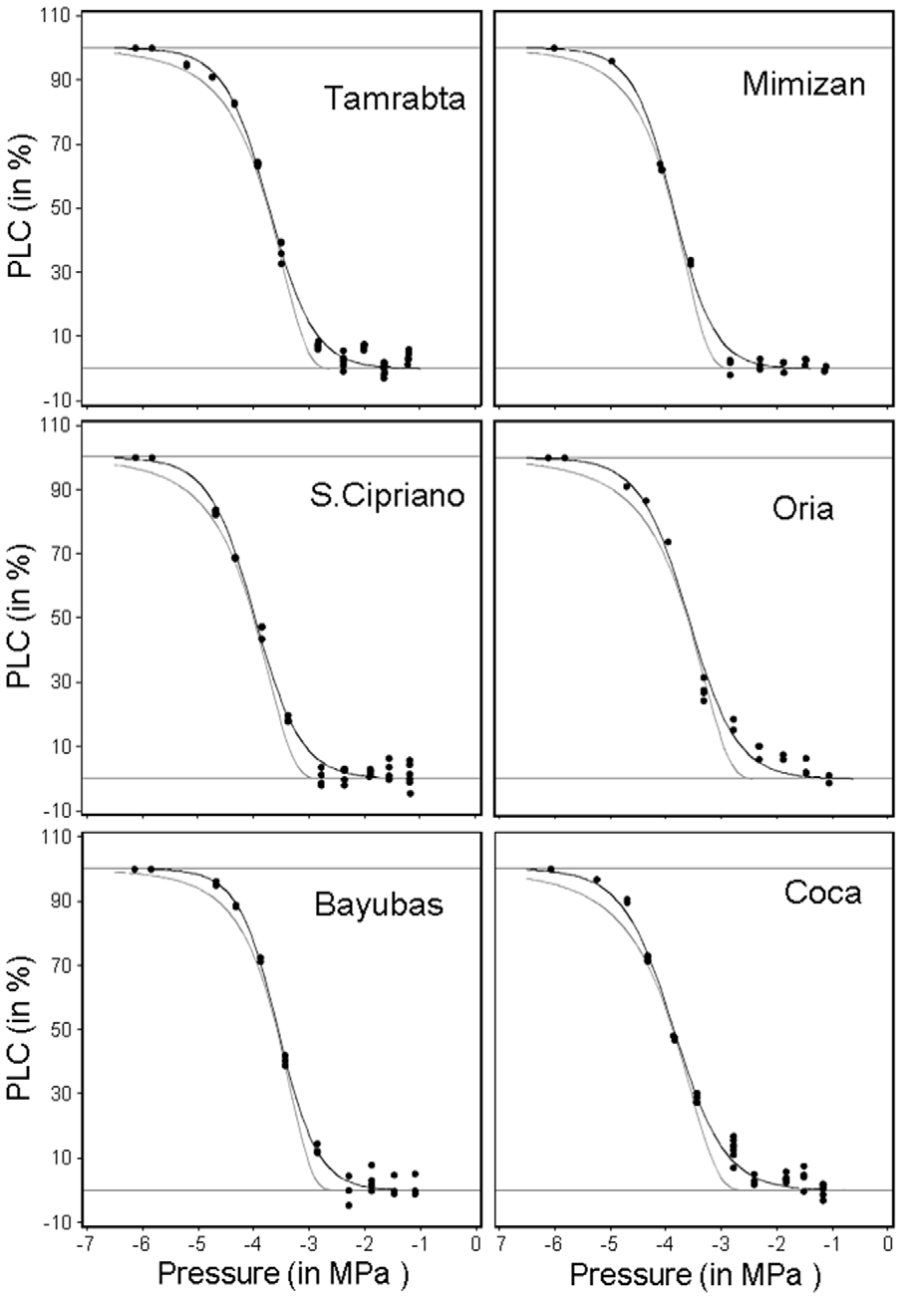
Vulnerability curves of one genotype for each studied population. Black dot are the raw measure of percent of loss of conductance (PLC in %) along the negative pressure gradient (in MPa). The grey line is the Weibull reparameterized model and the black line is the sigmoid reparameterized Model.

Cavitation resistance was measured on 240 genotypes (6 populations * 8 families * 5 offsprings), with the Cavitron technique [21], [27], [28]. Centrifugal force was used to establish negative pressure in the xylem and to provoke water stress-induced cavitation, using a custom-built honeycomb rotor (Precis 2000, Bordeaux, France) mounted on a high-speed centrifuge (HS18, MSE Scientific, London, UK). This technique enables measurement of the hydraulic conductance of a branch under negative pressure. Xylem pressure (*P*_x_) was first set to a reference pressure (−0.5 MPa) and maximal conductance (*k*_max_) was determined by measuring the flux of a reference ionic solution (10 mmol dm^−3^ KCl and dm^−3^ mmol dm^−3^ CaCl_2_ in deionized water) through the sample. The centrifugation speed was then set to a higher value for 3 min to expose the sample at a more negative pressure. Conductance (*k_i_*) were measured 4 times for each step, and the average was used to compute percent of loss of xylem of conductance (PLC in %) following PLC = 100 (1−*k_i_*/*k*_max_). The procedure was repeated for at least eight pressure step with a −0.5 MPa step increment until PLC reached at least 90%. The percent loss of xylem conductance as a function of xylem pressure (MPa) represents the sample's vulnerability curve (Figure 2). Rotor velocity was monitored with a 10 rpm resolution electronic tachymeter (A2108-LSR232, Compact Inst, Bolton, UK) and xylem pressure was adjusted to about ±0.02 MPa. We used Cavi_soft software (version1.5, University of Bordeaux) for conductance measurements and computation of all vulnerability curves (VCs). The 10,800 measurements of conductance were performed at the new high-throughput phenotyping platform for hydraulic traits (CavitPlace, University of Bordeaux, Talence, France).

Based on a sensitivity analysis (data not shown) and graphical checking (Figure 2), we retained the reparameterized sigmoid function fitted to the conductance data (*k_i_*) (see [29] and [30] for an exhaustive review) rather than the Weibull model, for determination of the pressure at which the sample lost 50% of its conductance (*P_50_*) and the slope of the curve at *P_50_* (*S_50_*).
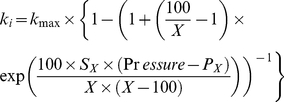
(1)where *k*_max_ is the highest conductance measured for each sample (equivalent to *k*_sat_ in the original Ogle's model), *k_i_* is the mean conductance at a given pressure concerned, *X* is the percentage loss of conductance of interest (in %), *P_X_* (in MPa) is the pressure inducing *X*% loss of conductance and *S_X_* (in MPa.% ^−1^) is the slope of the tangent at the *P_X_* abscissa point on the curve. Analysis has been performed for pressures and slopes corresponding to *X = *12, 50 and 88 % loss of conductivity (*P*_12_, *P*_50_, *P*_88_, *S*_12_, *S*_50_ and *S*_88_ respectively).

### Carbon isotope ratio and growth measurement

Carbon isotope ratio (*δ* ^13^C in %o) was obtained as previously described [32], [33]. Needles of the growth unit used for cavitation analysis were harvested and 20 needles were sampled at random. The needles were dried and ground to a powder and 3 mg sample was analyzed with an isotope ratio mass spectrometer (FISONS Isochrom, Manchester, UK) at INRA facility in Reims (France). Total height was measured at the ages of two (2004) and three (2005) years, on the same six populations and eight families, for all 15 blocks. The annual increase in height (Δ_h_) was calculated as the difference between these two measurements (in 2004 and 2005).

### Quantitative genetic analysis

Genetic analysis was conducted with the following mixed model:
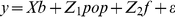
(2)where ***y*** is the vector of observation for a trait, ***b*** is the vector (number of block) of fixed block effects, ***pop*** is the vector (number of populations) of random population effects, ***f*** is the vector (number of mother trees) of the random genetic effects of mother tree within the population, **ε** is the vector (number of individuals) of residuals, **X** is called the design matrix, **Z_1_** and **Z_2_** are the incidence matrices linking the observations to the effects. A variance was fitted for each random effect: 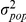 is the genetic variance between populations, 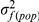 is the genetic variance between mother trees nested within a population and 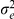
is the residual variance.

Variance or covariance components were estimated by the restricted maximum likelihood (REML) method, assuming a normal distribution of the random effects. The significance of variance components were tested using log-likelihood ratio tests. We included population as a random effect to draw inference at species levels [34] and to obtain an unbiased estimate of heritability and genetic population differentiation [35]. The normality, identity and independency of residuals of each trait were graphically checked by plotting studentized marginal and conditional residuals (available on request), which confirmed that the data match with the assumption of mixed model. We estimated narrow-sense heritability as follows:
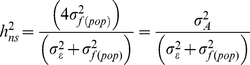
(3)where 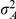 is the within-population additive variance. In our study, 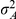 was estimated by 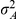 = 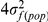 as trees from the same family were presumed to be half-sibs (open-pollinated seeds). We did not include the population effect in the heritability calculation, because natural selection appeared to occur within each population [36]. The standard deviation of heritability was calculated with the equations of delta method (see Appendix in [37]).

Variance components were standardized by the trait mean [38] as follows, CV_X_ = 100√(Variance)/Mean_X_ where X is the trait considered, and CV is the coefficient of variation. Each variance component is expressed with a CV (CV_A_: additive coefficient of variation; CV_BP_ (V_BP_ = 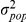): coefficient of variation between populations; CV_P_: phenotypic coefficient of variation; CV_R_: residual coefficient of variation). The variance of each component was extracted from the asymptotic covariance matrix. The significance of mean population difference was estimated using the same model (Eq. 2) with a proc GLM with a Student-Neuman-Keuls post hoc test.

### Correlation between traits

To facilitate interpretation of correlation, negative value of *P*_50_ and *δ* ^13^C were converted from negatives to positives. Genetic correlations between traits were evaluated by calculating Pearson's coefficient on the family Best Linear Unbiased Predictor estimation (BLUP, for additional information see [37] p745). BLUP estimation ensures that data are corrected for block effect. We will refer to these correlations as genetic correlations. For phenotypic correlation, all Pearson correlations were computed over the BLUP family plus BLUP population and the grand-mean.

### Estimation of population differentiation

The estimate of phenotypic differentiation between populations, *Q_ST_* [39], was calculated as
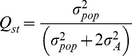
(4)Putatively neutral nuclear microsatellites (nuSSRs) were used to account for genetic differentiation (*F_ST_*) caused by demographic and other processes not related to selection (e.g., genetic drift resulting from geographic isolation or population expansion). Eight polymorphic nuSSRs were selected from those previously developed by [40] (NZPR413, NZPR1078, ctg64), [41], [42] (ctg275, FRPP91, FRPP94, ITPH4516) and [43] (A6F03). The markers were selected to be evenly distributed over the various linkage groups of the maritime pine genetic linkage map, with, at least, 4 alleles and multiplexing capacity. Genotyping was performed on genomic DNA isolated from the needles of 20 to 30 individuals from each of the six selected populations, as previously described [44], [45]. We used *F*_ST_ (which is estimated from the allelic frequency) rather than *R*_ST_ (which also takes into account allele size) because genetic drift affects allele frequency but not mutation rate. *F_ST_* values for each locus were estimated with Genepop [46] using the framework developed by [47] adapted for SSR data [48].

### *F*_ST_ and *Q*_ST_ comparison

For the comparison of *Q_ST_* and *F_ST_*, to disentangle the effects of genetic drift from those of selection, we develop a new test to avoid previously reported limitations [49], [50], [51], [52] and allow to test *Q_ST_* >*F_ST_* and *Q_ST_* <*F_ST_*. We explicitly derive the *F*_ST_ and *Q*_ST_ distribution using in both case a parametric bootstrap as follows.
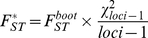
(5)
*F*_ST_^*^ is a parametric bootstrap replicate of *F*_ST_. First, nuSSR loci were randomly resampled with replacement, to estimate the sampling variance of *F_ST_*. Each of this *F_ST_* replicate (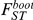) value was then multiplied by a random number drawn from the Lewontin-Krakauer distribution, a chi-squared distribution with a number of degree of freedom equal to the number of loci minus 1, divided by degree of freedom equal to the number of loci minus 1. This distribution has been shown to take into account most of the deviation from the neutral model due to demographic history [51], [53]. We will refer to this distribution of *F*_ST_^*^ as the “drift distribution”.

We estimated the sampling variance of *Q*_ST_ (Eq 4), by simulating the distribution of each variance component (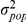,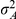) with a parametric bootstrap [50], using the Satterthwaite approximation [54]. This distribution is highly conservative and takes into account the deviation from homogeneity of variance [54].
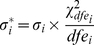
(6)

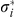
 is a parametric bootstrap replicate of variance component of *i* factor. It is obtained by multiplying 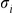 (observed variance component) by a chi-square distribution scaled with an “effective” degree of freedom (*dfe_i_*).
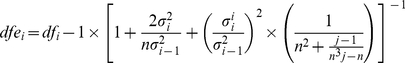
(7)
*dfe*_i_ is the effective degree of freedom for the variance component of *i* factor. *df*_i_ is the observed degree of freedom. 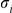 is observed variance component due to *i* factor. 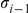 is observed variance component due to *i-1* factor (nested or residuals factor). *n* is the total size of the sample. *j* is the number of level of factor *i*. We calculate a *Q*_ST_^*^ for each replicate from 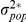 and 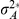 (Eq. 6). We will refer to this distribution of *Q*_ST_^*^ as the “phenotypic distribution”, although *Q*_ST_ is a standardized measurement of additive genetic variance between populations.

Finally, we compared the *F*_ST_^*^ and Q_ST_^*^ distributions, using nonparametric and free distribution two-sample test for equality of the 2.5 and the 97.5 quantile (see [55] for theoretical proof) with a Bonferroni correction for multiple comparisons. We also applied a studentized bootstrap, which gave similar results but required more computation time [56], [57], [58]. All the analyses were performed with SAS version 9.2. Codes are available on request.

## Results

### 1. Between-population variation

For each population, vulnerability curves showed similar sigmoid shape with the air-entry (*P*_12_) around −3.25±0.006 MPa (see Figure 2). Linear curves were discarded from the analysis [59]. The between-population effect (V_BP_) was significant for δ^13^C and Δ_h_ but not for *P*_50_ (Table 2). Similarly, no difference was found for the other cavitation resistance-related traits (*S*_12_, *S*_88_, *S*_50_, *P*_12_, *P*_88_, data not shown). Cavitation resistance-related traits had much lower coefficients of variation than Δ_h_ (Table 2). This was particularly true for the between-population coefficient of variation (CV_BP_ = 1% and 18% for *P*_50_ and Δ_h_, respectively). It should be noted that CVs for *δ* ^13^C are not comparable with those of other traits, because they are estimated relative to a standard [60] and are therefore independent of scale change but not of origin. The fixed block effect was significant for all the traits studied, indicating that some of the environmental variation was taken into account by the experimental design.

**Figure 3:**
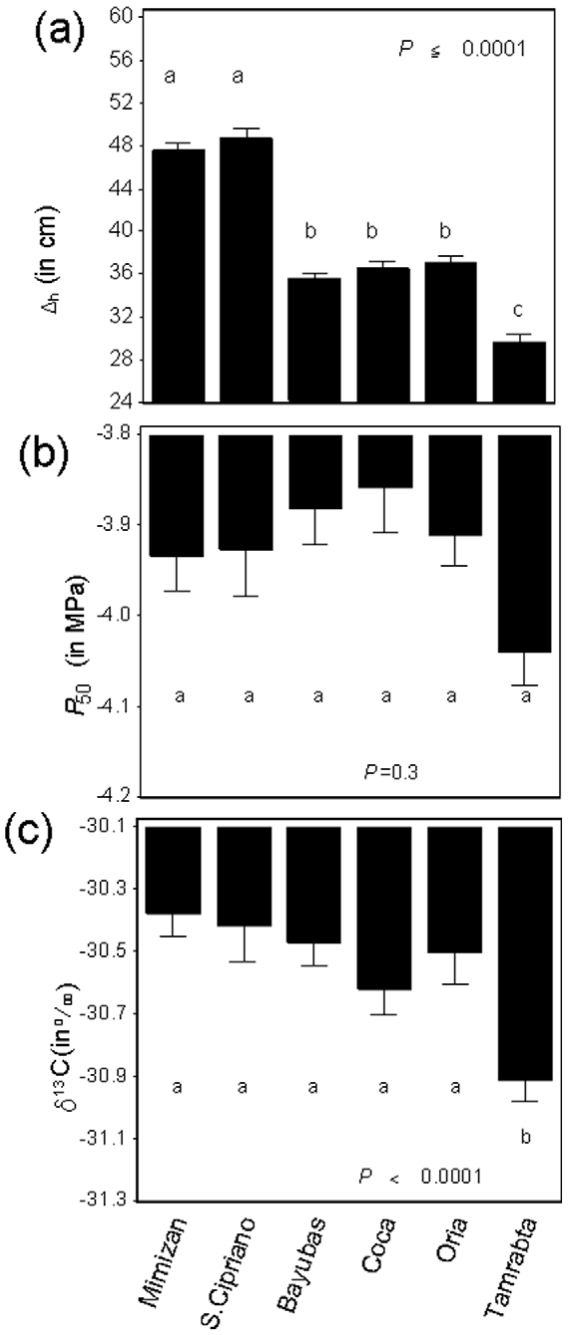
Mean values of height increment (Δ_h_, (a)) (n = 297 per population). Mean values of cavitation resistance (*P*_50_, (b)) and carbon isotope composition (δ^13^C, (c)) for each studied population (n = 40 per population). The error bars represents the standard errors. Different letters indicate significant differences between populations at α = 0.05.

The populations from the wettest areas (Mimizan and San Cipriano) had the highest Δ_h_ values (Figure 3a), whereas Tamrabta population (from Morocco) presented the lowest value. Iberian populations from very different climatic areas (Coca, Bayubas, Oria) had intermediate values, with no detectable trend as a function of environmental aridity.

No significant difference between populations was detected for *P*_50_ (Figure 3b), although Tamrabta surpassed the other populations and was the most cavitation-resistant population. Tamrabta also presented the lowest *δ* ^13^C value (Figure 3c), demonstrating a significantly lower water-use efficiency than the other populations, all other populations presented similar *δ* ^13^C values.

**Table 2:** Variance components (V_P_, V_BP_, V_A_, V_R_), narrow-sense heritability (h^2^_ns_), coefficient of variation (CV_P_, CV_A_, CV_BP_, CV_R_) and population differentiation (*Q*_ST_) for all studied maritime pine populations.

Traits	V_P_	V_BP_	V_A_	V_R_	h^2^_ns_±SE	CV_P_	CV_A_	CV_BP_	CV_R_	Q_ST_
*P*_50_	0.067	0.002 ns	0.028*	0.058	0.438±0.18	6.6	4.4	1	6.2	0.027
δ13C	0.284	0.030 **	0.059*	0.269	0.213±0.10	1.7^a^	0.8^a^	0.6^a^	1.7^a^	0.197
Δ_h_	112.7	55.0 ***	40.96***	102.5	0.363±0.06	26.9	16.2	18.8	25.7	0.188

**h^2^_ns_** is the narrow-sense heritability and **SE** is the standard error of heritability, **V_P_** is the phenotypic genetic variance, **V_A_** is the additive genetic variance, **V_BP_** is the between-population variance, **V_R_** is the residual variance. **CV_A_** is the variation coefficient of additive variance after adjustment for the block effect. **CV_P_** is the variation coefficient of phenotypic variance after adjustment for the block effect. **CV_R_** is the residual coefficient of variation. **CV_BP_** is between-population coefficients of variation. **Q_ST_** is the genetic quantitative variation between populations (Spitze, 1993). The significance of random effects is indicated after each variance estimator: ^ns^ *P* > 0.05, ^*^ *P*<0.05, ^**^ *P*<0.01, ^***^ *P*<0.001. ^a^ CVs for δ^13^C are not comparable with other traits as they are estimated relative to a standard.

### 2. Within-population variation

Heritabilities and normalized measurements of trait dispersion (i.e. CVs) were estimated to evaluate the within-population additive variance, evolvability (through the analysis of CV_A_) and micro-environmental sensitivity (through the analysis of CV_R_) (Table 2). Narrow-sense heritability (h^2^_ns_) for *P*_50_ was higher (0.44±0.18) than those estimated for *δ* ^13^C (0.21±0.10) and Δ_h_ (0.35±0.06), showing that cavitation resistance was genetically controlled, although the standard error was high, probably due to the small number of progenies per mother tree analyzed. The CVs of *P*_50_ and Δ_h_ presented contrasting patterns, with a lower coefficient of additive variation for *P*_50_ (CV_A_ = 4.4%) than for Δ_h_ (CV_A_ = 16.2%), suggesting limited evolvability of *P*_50_.

### 3. Evolutionary forces driving population differentiation

**Figure 4:**
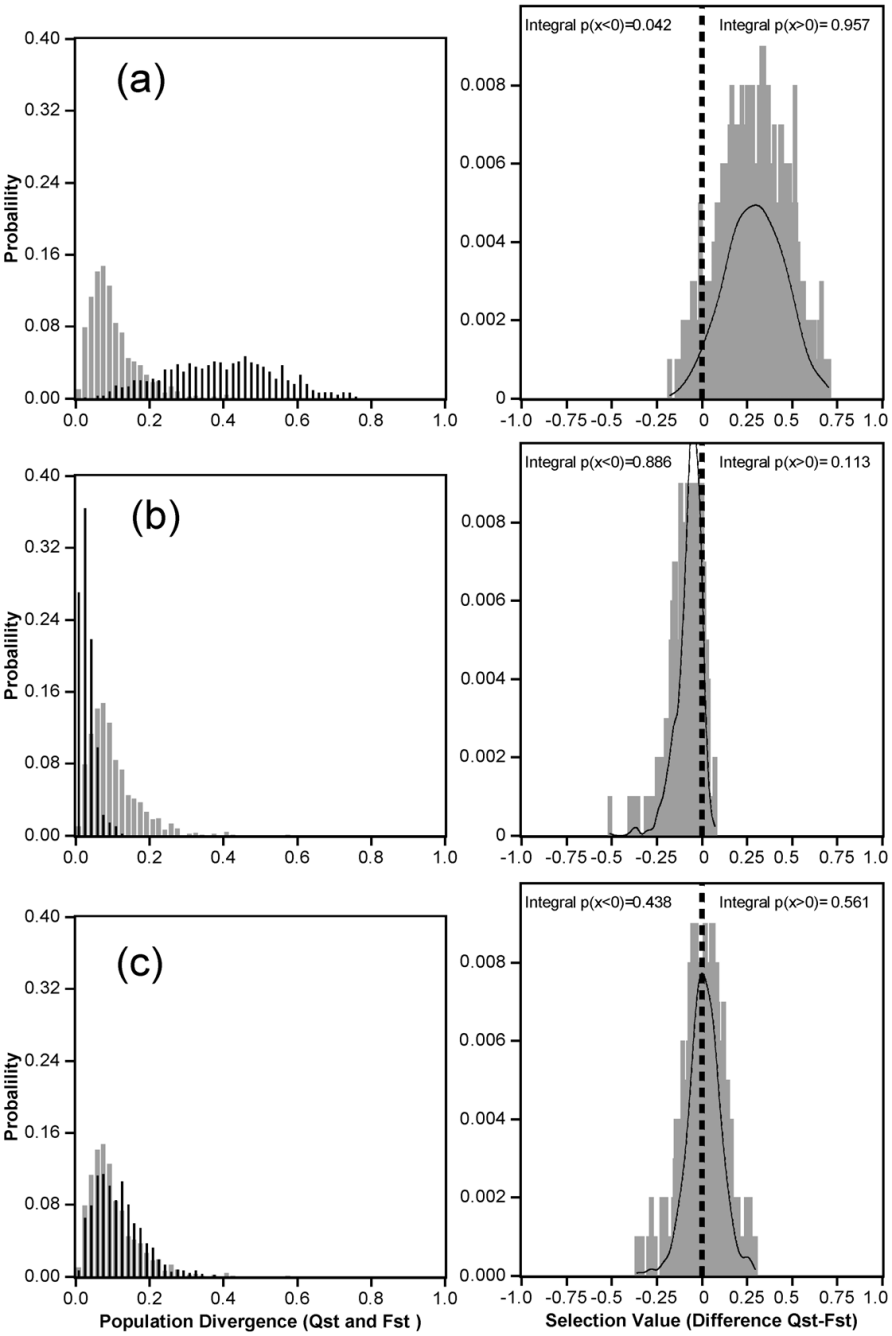
Comparison between *F*_ST_ (histogram in gray) and *Q*_ST_ (histogram in black) distributions for growth rate (Δ_h_, (a)), cavitation resistance (*P*_50_, (b)) and carbon isotope composition (*δ* ^13^C, (c)) in the left panel. The observed distribution (gray histogram) and the kernel density (black curves) of the *Q*_ST_-*F*_ST_ difference are represented in the right panel for each trait. On the right panel, we also show the integral probability of the distribution (using the kernel density estimator) above (see “Integral p(x>0)” on the right panel) and below (see “Integral p(x<0)” on the right panel) zero (marked with the tick and dotted line).

*Q*_ST_ and *F*_ST_ comparisons have three possible outcomes [39]: (i) if *Q*_ST_ > *F*_ST_, the degree of differentiation for quantitative traits exceeds that attainable by genetic drift alone (ii) if *Q*_ST_ and *F*_ST_ are not significantly different, the observed degree of differentiation for quantitative traits could have been reached by genetic drift alone, and (iii) if *Q*_ST_<*F*_ST_ the observed degree of differentiation is lower than expected from genetic drift alone. Consistent with previous reports [50], [61] , we found that *F*_ST_^*^ and *Q*_ST_^*^ presented skewed distributions (Figure 4). Only Δ_h_ and *P*_50_ had a *Q*_ST_^*^ distribution different from the *F*_ST_^*^ distribution (*P* = 0.003 and *P* <0.0001 respectively). For, *δ* ^13^C, the difference between *Q*_ST_^*^ and *F*_ST_^*^ values was centered on 0 (see Figure 4c right panel), and it was therefore not possible to distinguish between drift and selection (*P* = 0.88). Conversely, differences between the *Q*_ST_^*^ and *F*_ST_^*^ distributions for *P*_50_ were centered on -0.18, suggesting that the studied populations were less differentiated than would be expected in the presence of drift alone (Figure 4b), which means that natural selection favored the same mean phenotype in different populations (consequence of uniform selection) . For Δ_h_, the difference between *Q*_ST_^*^ and *F*_ST_^*^ distributions was centered around 0.27, suggesting that the studied populations displayed more differentiation than would be expected with drift alone (Figure 4c) which is interpreted as a consequence of diversifying selection.

### 4. Correlation between traits

We found a significant positive phenotypic correlation between absolute value of *P*_50_ and *δ* ^13^C at the phenotypic level (r = 0.30, *P* = 0.035 see Figure S1), indicating that the more cavitation-resistant genotypes tended to be less water-use efficient. However, this relationship was not significant at the genetic level (r = 0.14, *P* = 0.320). No relationship between *P*_50_ and Δ_h_ was found at either phenotypic or genetic level. A significant negative phenotypic correlation between Δ_h_ and *δ* ^13^C was detected (r = −0.68, *P*<0.0001). This correlation was barely significant at the genetic level (r = −0.29, *P* = 0.053).

## Discussion

We reliably estimated for the first time the genetic variability of cavitation resistance, a functional trait that allows plants to survive under severe drought. We also provided evidence of natural selection acting on this trait. These results were based on the greatest number of genotypes ever measured to date in an experimental design (Table S1 and supplementary references). Despite the high level of variation of cavitation resistance between species [15], we detected no significant differences between maritime pine populations from a wide range of environments. Moreover, the between-population variability of cavitation resistance was significantly lower than would be expected under a hypothesis of genetic drift alone (*Q*_ST_- *F*_ST_ comparison). We can therefore reject the hypothesis of diversifying selection. We suggest instead that uniform selection has shaped the phenotypic variability of this trait. Uniform selection could be seen as a stabilizing selection acting within each population with the same selection optimum in each population despite the steep climatic gradient [62], [63]. Conversely, growth and water-use efficiency displayed different patterns and were found to be subject to strong diversifying selection and genetic drift, respectively. Quantitative genetics analysis also showed that cavitation resistance presented a significant heritability, higher than that estimated for growth and water-use efficiency. This is the first evidence of uniform selection in woody plants and the underlying mechanisms are discussed below from a micro-evolutionary point of view.

### Intra- vs. interspecific variability of cavitation resistance

Despite the steepest climatic gradient (precipitation ranging from 400 to 1,600 mm in the sampled populations) and strong phylogeographic structure between the six studied populations [22], [64]. Very low between-population variance for cavitation resistance were found (CV_BP = _1%). The few studies published to date (reviewed in Table S1) tended to skim over the issue of intraspecific variation of cavitation resistance in provenance or progeny trials and reported little or no difference between populations [65], [66]. As these studies were not designed to assess the genetic component of phenotypic variation, further investigations are required, to generalize our finding to other species. In addition, phenotypic variation for cavitation resistance was low (CV_P_ = 6.6%), but consistent with the range reported for wood properties of maritime pine, such as mean ring density, lignin content and fiber morphology ([67], [68] Lamy, unpunblished data). However, we are lacking information about the intraspecific variation of pit pair anatomical traits that are known to be implicated in cavitation resistance [69], [16]. The low within-species variability for cavitation resistance is remarkable (*P*_50_ = −3.93±0.04 MPa, estimated over the whole dataset), given that substantial variability has been described between species. For instance, Delzon et al (2010) showed that cavitation resistance ranged from −3 to −12 MPa in a sample of 40 coniferous species. This variability was interpreted as the effect of natural selection rather than phylogenetic legacy [15].

In contrast, the population differentiation observed for growth and water-use efficiency (WUE) was significant and consistent with previous results for this species [70]. The Moroccan population had the lowest WUE, consistent with previous findings based on both gas exchange measurements and carbon isotope discrimination [71], [72], [73], [74]. In a provenance trial carried out in south-western France, this Moroccan population displayed lower stomatal sensitivity to water stress (delayed stomatal closure), leading to greater water loss throughout the summer period and a lower WUE (as reflected by carbon isotope composition). Genotype × environment interaction could potentially alter differences between populations [75], [76]. Our results therefore require confirmation in provenance trials carried out in drier climates.

### Relationships between traits

The weak but significant positive correlation found between absolute value of cavitation resistance and water-use efficiency (carbon isotope composition) suggested that drought-tolerant genotypes had lower water-use efficiency. In dry environments, genotypes that allocate more carbon to the construction of cavitation-resistant wood in order to avoid runaway embolism might be able to maintain higher stomatal conductance and hydraulic conductance at low leaf water potential, resulting in a decrease in water-use efficiency. Our results are consistent with previous findings [77] of a strong and positive relationship between these two traits in two cedar species. However, little or no correlation has generally been reported [78], [79], [80]. The negative correlation between carbon isotope composition and growth has been reported in previous studies [77], [81], [82], assuming that growth is a function of carbon assimilation and carbon isotope composition is an index of retrospective gas exchanges.

### Evidence of uniform selection for cavitation resistance

The phenotypic distribution of cavitation resistance was significantly lower than the expected distribution under the drift hypothesis. This may be interpreted as a consequence of uniform selection (also called homogenous, spatially homogenizing, convergent selection, uniform stabilizing selection or stabilizing selection across population). This inference (*Q*_ST_<*F*_ST_) may result from an underestimation of *Q*_ST_ variance [52], leading to a false positive result. However, the *F*_ST_ estimate was more than five times greater that the *Q*_ST_ estimate. We thus believe that this difference is biologically meaningful and not due to a statistical artifact [52]. The robustness of this result is, also, supported by fourth lines of evidence: (i) the different patterns obtained for growth and WUE in the same experimental design. (ii) Selection procedure of population (see methods) increase the probability to find diversifying selection because we selected extreme populations in term of climatic origin and evolutionary history (different mitotypes and chlorotypes, see [64]), consequently uniform selection could not be interpreted as a sampling bias. (iii) Wood density (measured by X ray on the same data set) showed exactly the same pattern (Lamy, unpunblished). (iv) Willson et al (2008) showed from interspecific data with narrow taxon sampling (limited to the *Juniperus* genus), that cavitation resistance gave strong phylogenetic conservatism, suggestive of uniform selection for the maintenance of ancestral traits.

For growth, diversifying selection was highlighted by *Q*_ST_ being greater than *F*_ST_ [83]. For WUE, we detected no signature of selection, as the phenotypic and drift distributions did not differ significantly, as previous reported by [84] for *Quercus suber* (*P*_ST_/*F*_ST_ comparison). However, the distribution (*Q*_ST_-*F*_ST_) of this trait was shifted to the right (integral probability above 0 > integral probability below 0, Figure 4c right panel), suggesting that diversifying selection was slightly more pronounced than drift.

### What are the mechanisms behind “uniform selection” for cavitation resistance?

The causal mechanisms underlying uniform selection or leading to evolutionary stasis are not well understood [63], [85], [86], [87], [88]. We discuss here only the processes most likely to account for the observed pattern (*Q*_ST_<*F*_ST_).

#### Weak molecular variation

Lethal or sublethal mutations may limit the variability of the genes they affect, thereby controlling trait variation. In conifers, cavitation resistance is known to be determined by xylem anatomy, including, in particular, the characteristics of intertracheid pits [16], [69]. As knowledge about the nucleotide diversity of different functional categories of genes accumulates, it may become possible to test the hypothesis that genes involved in intertracheid pit formation (once these genes have been identified) display lower levels of diversity.

#### Genetic constraints

If selection acts on a trait that is negatively correlated with another trait (or traits) also under selection, than the decrease of rate of evolution for the first trait is proportional to the strength of the correlation [94]. A multi-trait approach could be used to explore this hypothesis indirectly [95], but could fail if the trait is canalized.

#### Canalized trait

This hypothesis suggest that cavitation resistance is canalized to buffer the variation of this key hydraulic trait against all kinds of disturbance, being of genetic (mutation, hybridization, recombination) and/or environmental nature [89], [90], [91]. Emergent properties of molecular networks could buffer molecular variability [92], to maintain phenotypic function, in accordance with the robustness theory [93]. In zoology, dipterian wings shape or centroid size (or mammalian body temperature) are the best known cases of canalized traits [90]. Indeed they reported a similar wing shape between species despite a great variability of climatic niche and a low additive genetic variance between populations for this trait. Except for leaf shape in *Arabidopsis thaliana*, there is no evidence of canalized trait in plants nowadays. For cavitation resistance, two arguments lead us to consider canalization as the most likely mechanism: (i) low additive genetic variance between populations (*V*_BP_) and (ii) the similarity of cavitation resistance values among all the *Pinus* species [69].

### Variance component analysis

Variance component analysis for cavitation resistance resulted in the first estimates of the heritability of this trait (h^2^_ns_ = 0.4) and its additive coefficient of variation (CV_A = _4.4%). These values suggest that this trait may respond to truncation selection frequently practiced in breeding. However, for a given selection intensity, genetic gain for cavitation resistance would be limited by the low additive variance, although long-term artificial selection experiments have shown that quantitative traits have a non negligible mutational variance [91], [96], [97], which could supply further additive variance at each generation. However, due to the small number of half-sib families and progenies within each family, which could inflate the value for heritability [37], this estimate should be interpreted with caution. Additional studies with a larger sample size are required for further exploration of the genetic determinism of this hydraulically important trait.

The much higher CV_A_ value (16.2%) for height increment is consistent with previous reports and accounts for the genetic gain achieved for this trait over successive generations in breeding programs [98], [99], [100]. For δ^13^C, a previous study [31] reported a slightly lower heritability (h^2^_ns_ = 0.17 vs. 0.21 here), but with estimation based on a diallel cross of limited size, with a narrow genetic background restricted to 12 elite trees from south-western France.

### Future directions

Phenotypic variation is a fundamental prerequisite for evolution because natural selection acts on phenotype. Adaptation and evolution via natural selection requires the presence of genetic variation among individuals in a population upon which natural selection can act. Intra-population genetic variability can thus be seen as the fuel for future adaptation. However, an environmentally induced shift in phenotype is also a major component of the variation we see in nature. Recent studies [80], [101] showed a weak but significant phenotypic variability for cavitation resistance. Our results suggest that this between populations variability might be under environmental control rather than genetic determinism. These considerations call for more research (ongoing) aiming at quantifying the *in situ* phenotypic variability of cavitation resistance and the extent of phenotypic plasticity using provenance trials installed under different edapho-climatic environments. Further studies are also being pursued to dissect the genetic architecture of cavitation resistance to determine the number, map location and effects of Quantitative Trait Loci controlling part of the variation of this trait.

## Supporting Information

Figure S1 **Genetic (right panel) and phenotypic (left panel) correlation between traits.** For ease of interpretation, we have converted all the negative values to positive values (P_50_, δC^13^). For the genetic correlation, all Pearson correlations (r) were computed over the best linear unbiased prediction (BLUP) (n = 48 for P_50_, δ^13^ and n = 151 for Δh). For phenotypic correlation, all Pearson correlations were computed over the BLUP family plus BLUP population and the grand-mean, to ensure that the order of degree of freedom remained the same and the block effects are removed. P_50_, pressure at 50 % loss of conductivity in MPa, Δh the annual increment between 2004 and 2005, in mm, δ^13^C is the isotope discrimination for carbon 13 in ‰.
(TIF)

Table S1 **Review of intraspecific studies for cavitation resistance estimated using** ***P*** **_50_ or related parameters (as indicated in the table).** Npop: number of populations used, Nind: number of individuals per population used to assess cavitation resistance. The table is divided in two parts, the first part corresponds to provenance or progeny trials, and the second to “*in situ*” studies.
(DOC)

Table S2 **Result of the principal component (PC) analysis (PCA) for climatic data of** ***Pinus pinaster*** **populations (listed in the methods section, n = 763).** Contributions to the first, second, third and fourth axes are indicated for each variable (PC1, PC2, PC3, PC4). The eigenvalues of PC1 = 7.65, PC2 = 3.059, PC3 = 0.97, PC4 = 0.86. *W* is mean wet ground days (days). *I* is mean Martonne's index (P_i_/(T_a_+10)). *P_i_* is the mean precipitation (mm.days^−1^). *C* is percent of cloud cover (%). *S* is the mean of wind speed (m.s^−1^). *V* is the water vapor pressure in air (hPa). *VPD* is the water vapor pressure deficit of air (hPa). *T*_min_ is the minimum temperature (°C). Δ_DT_ is the mean diurnal temperature range (°C). *T*_m_ is mean temperature (°C). *R_G_* is mean global radiation (W.m^2^). *T*_max_ is the maximum temperature (°C). *H* is mean soil water deficit (P_i_-ETP, in mm). *ETP* is mean Truc's potential evapotranspiration (mm).
(DOC)

## References

[1] Pearson RG (2006). Climate change and the migration capacity of species.. Trends in Ecology & Evolution.

[2] Thuiller W (2004). Patterns and uncertainties of species' range shifts under climate change.. Global Change Biology.

[3] Lindner M, Maroschek M, Netherer S, Kremer A, Barbati A (2010). Climate change impacts, adaptive capacity, and vulnerability of European forest ecosystems.. Forest Ecology and Management.

[4] Beniston M, Stephenson DB, Christensen OB, Ferro CAT, Frei C (2007). Future extreme events in European climate: an exploration of regional climate model projections.. Climatic Change.

[5] Breshears DD, Myers OB, Meyer CW, Barnes FJ, Zou CB (2009). Tree die-off in response to global change-type drought: mortality insights from a decade of plant water potential measurements.. Frontiers in Ecology and the Environment.

[6] Martinez-Meier A, Sanchez L, Pastorino M, Gallo L, Rozenberg P (2008). What is hot in tree rings? The wood density of surviving Douglas-firs to the 2003 drought and heat wave.. Forest Ecology and Management.

[7] Martinez-Vilalta J, Pinol J (2002). Drought-induced mortality and hydraulic architecture in pine populations of the NE Iberian Peninsula.. Forest Ecology and Management.

[8] Jentsch A, Kreyling J, Beierkuhnlein C (2007). A new generation of climate-change experiments: events, not trends.. Frontiers in Ecology and the Environment.

[9] McDowell N, Pockman WT, Allen CD, Breshears DD, Cobb N (2008). Mechanisms of plant survival and mortality during drought: why do some plants survive while others succumb to drought?. New Phytologist.

[10] Tyree MT (2003). The ascent of water.. Nature.

[11] Brodribb TJ, Bowman D, Nichols S, Delzon S, Burlett R (2010). Xylem function and growth rate interact to determine recovery rates after exposure to extreme water deficit.. New Phytologist.

[12] Brodribb TJ, Cochard H (2009). Hydraulic Failure Defines the Recovery and Point of Death in Water-Stressed Conifers.. Plant Physiology.

[13] Cochard H, Barigah ST, Kleinhentz M, Eshel A (2008). Is xylem cavitation resistance a relevant criterion for screening drought resistance among Prunus species?. Journal of Plant Physiology.

[14] Hacke UG, Sperry JS (2001). Functional and ecological xylem anatomy.. Perspectives in Plant Ecology Evolution and Systematics.

[15] Maherali H, Pockman WT, Jackson RB (2004). Adaptive variation in the vulnerability of woody plants to xylem cavitation.. Ecology.

[16] Pittermann J, Choat B, Jansen S, Stuart SA, Lynn L (2010). The Relationships between Xylem Safety and Hydraulic Efficiency in the Cupressaceae: The Evolution of Pit Membrane Form and Function.. Plant Physiology.

[17] Willson CJ, Manos PS, Jackson RB (2008). Hydraulic traits are influenced by phylogenetic history in the drought-resistant, invasive genus Juniperus (Cupressaceae).. American Journal of Botany.

[18] Jacobsen AL, Ewers FW, Pratt RB, Paddock WA, Davis SD (2005). Do xylem fibers affect vessel cavitation resistance?. Plant Physiology.

[19] Ribeiro MM, LeProvost G, Gerber S, Vendramin GG, Anzidei M (2002). Origin identification of maritime pine stands in France using chloroplast simple-sequence repeats.. Annals of Forest Science.

[20] Richardson DM (1998). Ecology and biogeography of Pinus..

[21] Cochard H, Damour G, Bodet C, Tharwat I, Poirier M (2005). Evaluation of a new centrifuge technique for rapid generation of xylem vulnerability curves.. Physiologia Plantarum.

[22] Bucci G, Gonzalez-Martinez SC, Le Provost G, Plomion C, Ribeiro MM (2007). Range-wide phylogeography and gene zones in Pinus pinaster Ait. revealed by chloroplast microsatellite markers.. Molecular Ecology.

[23] New M, Hulme M, Jones P (1999). Representing twentieth-century space-time climate variability. Part I: Development of a 1961-90 mean monthly terrestrial climatology.. Journal of Climate.

[24] New M, Hulme M, Jones P (2000). Representing twentieth-century space-time climate variability. Part II: Development of 1901-96 monthly grids of terrestrial surface climate.. Journal of Climate.

[25] New M, Lister D, Hulme M, Makin I (2002). A high-resolution data set of surface climate over global land areas.. Climate Research.

[26] Delzon S, Sartore M, Burlett R, Dewar R, Loustau D (2004). Hydraulic responses to height growth in maritime pine trees.. Plant Cell and Environment.

[27] Cai J, Hacke U, Zhang SX, Tyree MT (2010). What happens when stems are embolized in a centrifuge? Testing the cavitron theory.. Physiologia Plantarum.

[28] Cochard H (2002). A technique for measuring xylem hydraulic conductance under high negative pressures.. Plant Cell and Environment.

[29] Pammenter NW, Vander Willigen C (1998). A mathematical and statistical analysis of the curves illustrating vulnerability of xylem to cavitation.. Tree Physiology.

[30] Ogle K, Barber JJ, Willson C, Thompson B (2009). Hierarchical statistical modeling of xylem vulnerability to cavitation.. New Phytologist.

[31] Brendel O, Pot D, Plomion C, Rozenberg P, Guehl JM (2002). Genetic parameters and QTL analysis of delta C-13 and ring width in maritime pine.. Plant Cell and Environment.

[32] Farquhar GD, Oleary MH, Berry JA (1982). On the relationship between carbon isotope discrimination and the inter-cellular carbon-dioxide concentration in leaves.. Australian Journal of Plant Physiology.

[33] Farquhar GD, Richards RA (1984). Isotopic composition of plant carbon correlates with water-use efficiency of wheat genotypes.. Australian Journal of Plant Physiology.

[34] SAS II (2008). SAS/STAT® 9.2 User's Guide..

[35] Wilson AJ (2008). Why h^2^ does not always equal V_A_/V_P_?. Journal of Evolutionary Biology.

[36] Visscher PM, Hill WG, Wray NR (2008). Heritability in the genomics era - concepts and misconceptions.. Nature Reviews Genetics.

[37] Lynch M, Walsh B (1998). Genetics and analysis of quantitative traits: Sinauer Associates, Inc...

[38] Houle D (1992). Comparing evolvabiliy and variability of quantitative traits.. Genetics.

[39] Spitze K (1993). Population-Structure in Daphnia-Obtusa - Quantitative Genetic and Allozymic Variation.. Genetics.

[40] Chagne D, Chaumeil P, Ramboer A, Collada C, Guevara A (2004). Cross-species transferability and mapping of genomic and cDNA SSRs in pines.. Theoretical and Applied Genetics.

[41] Mariette S, Chagne D, Decroocq S, Vendramin GG, Lalanne C (2001). Microsatellite markers for Pinus pinaster Ait.. Annals of Forest Science.

[42] Mariette S, Chagne D, Lezier C, Pastuszka P, Baffin A (2001). Genetic diversity within and among Pinus pinaster populations: comparison between AFLP and microsatellite markers.. Heredity.

[43] Guevara MA, Chagne D, Almeida MH, Byrne M, Collada C (2005). Isolation and characterization of nuclear microsatellite loci in Pinus pinaster Ait.. Molecular Ecology Notes.

[44] Eveno E (2008). Drought adaptation in *Pinus pinaster*: diversity pattern and nucleotidic differentiation of candidats genes and phenotypic variability [PhD thesis,]..

[45] Eveno E, Collada C, Guevara MA, Leger V, Soto A (2008). Contrasting patterns of selection at Pinus pinaster Ait. drought stress candidate genes as revealed by genetic differentiation analyses.. Molecular Biology and Evolution.

[46] Rousset F (2008). GENEPOP ' 007: a complete re-implementation of the GENEPOP software for Windows and Linux.. Molecular Ecology Resources.

[47] Weir BS, Hill WG (2002). Estimating F-statistics.. Annual Review of Genetics.

[48] Michalakis Y, Excoffier L (1996). A generic estimation of population subdivision using distances between alleles with special reference for microsatellite loci.. Genetics.

[49] Le Corre V, Kremer A (2003). Genetic variability at neutral markers, quantitative trait loci and trait in a subdivided population under selection.. Genetics.

[50] O'Hara RB, Merila J (2005). Bias and precision in *Q*
_ST_ estimates: Problems and some solutions.. Genetics.

[51] Whitlock MC (2008). Evolutionary inference from *Q*
_ST_.. Molecular Ecology.

[52] Whitlock MC, Guillaume F (2009). Testing for Spatially Divergent Selection: Comparing *Q*
_ST_ to *F*
_ST_.. Genetics.

[53] Lewontin RC, Krakauer J (1973). Distribution of gene frequency as a test of theory of selective neutrality of polymorphisms.. Genetics.

[54] Satterthwaite FE (1946). An Approximate Distribution of Estimates of Variance Components.. Biometrics Bulletin.

[55] Kosorok MR (1999). Two-sample quantile tests under general conditions.. Biometrika.

[56] Boos DD (2003). Introduction to the bootstrap world.. Statistical Science.

[57] Field CA, Pang Z, Welsh AH (2008). Bootstrapping data with multiple levels of variation.. Canadian Journal of Statistics-Revue Canadienne De Statistique.

[58] Field CA, Welsh AH (2007). Bootstrapping clustered data.. Journal of the Royal Statistical Society Series B-Statistical Methodology.

[59] Cochard H, Herbette S, Barigah T, Badel E, Ennajeh M (2010). Does sample length influence the shape of xylem embolism vulnerability curves? A test with the Cavitron spinning technique.. Plant Cell and Environment.

[60] Brendel O, Le Thiec D, Scotti-Saintagne C, Bodenes C, Kremer A (2008). Quantitative trait loci controlling water use efficiency and related traits in Quercus robur L.. Tree Genetics & Genomes.

[61] Waldmann P, Garcia-Gil MR, Sillanpaa MJ (2005). Comparing Bayesian estimates of genetic differentiation of molecular markers and quantitative traits: an application to Pinus sylvestris.. Heredity.

[62] Leinonen T, O'Hara RB, Cano JM, Merila J (2008). Comparative studies of quantitative trait and neutral marker divergence: a meta-analysis.. Journal of Evolutionary Biology.

[63] Merila J, Crnokrak P (2001). Comparison of genetic differentiation at marker loci and quantitative traits.. Journal of Evolutionary Biology.

[64] Burban C, Petit RJ (2003). Phylogeography of maritime pine inferred with organelle markers having contrasted inheritance.. Molecular Ecology.

[65] Matzner SL, Rice KJ, Richards JH (2001). Intra-specific variation in xylem cavitation in interior live oak (Quercus wislizenii A. DC.).. Journal of Experimental Botany.

[66] Wang TL, Aitken SN, Kavanagh KL (2003). Selection for improved growth and wood quality in lodgepole pine: effects on phenology, hydraulic architecture and growth of seedlings.. Trees-Structure and Function.

[67] Bouffier L, Charlot C, Raffin A, Rozenberg P, Kremer A (2008). Can wood density be efficiently selected at early stage in maritime pine (Pinus pinaster Ait.)?. Annals of Forest Science.

[68] Pot D, Chantre G, Rozenberg P, Rodrigues JC, Jones GL (2002). Genetic control of pulp and timber properties in maritime pine (Pinus pinaster Ait.).. Annals of Forest Science.

[69] Delzon S, Douthe C, Sala A, Cochard H (2010). Mechanism of water-stress induced cavitation in conifers: bordered pit structure and function support the hypothesis of seal capillary-seeding.. Plant, Cell & Environment in Press.

[70] Gonzalez-Martinez SC, Alia R, Gil L (2002). Population genetic structure in a Mediterranean pine (Pinus pinaster Ait.): a comparison of allozyme markers and quantitative traits.. Heredity.

[71] Correia I, Almeida MH, Aguiar A, Alia R, David TS (2008). Variations in growth, survival and carbon isotope composition (delta C-13) among Pinus pinaster populations of different geographic origins.. Tree Physiology.

[72] Guehl JM, Ferhi A, Loustau D, Nguyen A (1993). Spatial and between-tree variability of cellulose delta13C in the wood of maritime pine trees.. Agricoltura Ricerca.

[73] Guehl JM, Fort C, Ferhi A (1995). Differential response of leaf conductance, carbon-isotope discrimination and water-use efficiency to nitrogen deficiency in Maritime Pine and Pedunculate Oak plants.. New Phytologist.

[74] Guyon JP, Kremer A (1982). Phenotypic stability of the height growth and daily kinetics of sap pressure and transpiration in the maritime pine (*Pinus pianster*).. Canadian Journal of Forest Research-Revue Canadienne De Recherche Forestiere.

[75] Alia R, Moro J, Denis JB (1997). Performance of Pinus pinaster provenances in Spain: interpretation of the genotype by environment interaction.. Canadian Journal of Forest Research-Revue Canadienne De Recherche Forestiere.

[76] Rehfeldt GE, Tchebakova NM, Parfenova YI, Wykoff WR, Kuzmina NA (2002). Intraspecific responses to climate in Pinus sylvestris.. Global Change Biology.

[77] Ducrey M, Huc R, Ladjal M, Guehl JM (2008). Variability in growth, carbon isotope composition, leaf gas exchange and hydraulic traits in the eastern Mediterranean cedars Cedrus libani and C. brevifolia.. Tree Physiology.

[78] Fichot R, Barigah TS, Chamaillard S, Le Thiec D, Laurans F (2010). Common trade-offs between xylem resistance to cavitation and other physiological traits do not hold among unrelated Populus deltoidesx Populus nigra hybrids.. Plant Cell and Environment.

[79] Maherali H, Walden AE, Husband BC (2009). Genome duplication and the evolution of physiological responses to water stress.. New Phytologist.

[80] Martinez-Vilalta J, Cochard H, Mencuccini M, Sterck F, Herrero A (2009). Hydraulic adjustment of Scots pine across Europe.. New Phytologist.

[81] Baltunis BS, Martin TA, Huber DA, Davis JM (2008). Inheritance of foliar stable carbon isotope discrimination and third-year height in Pinus taeda clones on contrasting sites in Florida and Georgia.. Tree Genetics & Genomes.

[82] Johnsen KH, Flanagan LB, Huber DA, Major JE (1999). Genetic variation in growth, carbon isotope discrimination, and foliar N concentration in Picea mariana: analyses from a half-diallel mating design using field-grown trees.. Canadian Journal of Forest Research-Revue Canadienne De Recherche Forestiere.

[83] Goudet J, Buchi L (2006). The effects of dominance, regular inbreeding and sampling design on *Q*
_ST_, an estimator of population differentiation for quantitative traits.. Genetics.

[84] Ramirez-Valiente JA, Lorenzo Z, Soto A, Valladares F, Gil L (2009). Elucidating the role of genetic drift and natural selection in cork oak differentiation regarding drought tolerance.. Molecular Ecology.

[85] Brent Burt D (2001). Evolutionary stasis, constraint and other terminology describing evolutionary patterns.. Biological Journal of the Linnean Society.

[86] Caruso CM (2004). The quantitative genetics of floral trait variation in Lobelia: Potential constraints on adaptive evolution.. Evolution.

[87] Caruso CM, Maherali H, Mikulyuk A, Carlson K, Jackson RB (2005). Genetic variance and covariance for physiological traits in Lobelia: Are there constraints on adaptive evolution?. Evolution.

[88] Caruso CM, Maherali H, Sherrard M (2006). Plasticity of physiology in Lobelia: Testing for adaptation and constraint.. Evolution.

[89] Hansen TF, Alvarez-Castro JM, Carter AJR, Hermisson J, Wagner GP (2006). Evolution of genetic architecture under directional selection.. Evolution.

[90] Hansen TF, Houle D, Pigliucci M, Preston K (2004). Evolvability, stabilizing selection, and the problem of stasis.. Evolutionary Biology of Complex Phenotypes.

[91] Le Rouzic A, Carlborg O (2008). Evolutionary potential of hidden genetic variation.. Trends in Ecology & Evolution.

[92] Fu J, Keurentjes JJB, Bouwmeester H, America T, Verstappen FWA (2009). System-wide molecular evidence for phenotypic buffering in Arabidopsis.. Nature Genetics.

[93] Kitano H (2004). Biological robustness.. Nature Reviews Genetics.

[94] Kruuk LEB, Merila J, Sheldon BC (2001). Phenotypic selection on a heritable size trait revisited.. American Naturalist.

[95] Martin G, Chapuis E, Goudet J (2008). Multivariate Q(st)-F-st Comparisons: A Neutrality Test for the Evolution of the G Matrix in Structured Populations.. Genetics.

[96] Houle D, Morikawa B, Lynch M (1996). Comparing mutational variabilities.. Genetics.

[97] Moose SP, Dudley JW, Rocheford TR (2004). Maize selection passes the century mark: a unique resource for 21st century genomics.. Trends in Plant Science.

[98] Bouffier L, Raffin A, Kremer A (2008). Evolution of genetic variation for selected traits in successive breeding populations of maritime pine.. Heredity.

[99] Kremer A (1979). Genetic control of height increment in Pinus pinaster, annual rhythm and interannual behaviour.. Comptes-rendus du 104eme Congres National des Societes Savantes.

[100] Rweyongeza DM, Yeh FC, Dhir NK (2005). Heritability and correlations for Biomass production and allocation in white spruce seedlings.. Silvae Genetica.

[101] Herbette S, Wortemann R, Awad H, Huc R, Cochard H (2010). Insights into xylem vulnerability to cavitation in *Fagus sylvatica* L.: phenotypic and environmental sources of variability.. Tree Physiology.

