# Supplementary figures and images for: Uniform Selection as a Primary Force Reducing Population Genetic Differentiation of Cavitation Resistance across a Species Range
Source: PLoS One. 2011 Aug 12;6(8):e23476. doi: 10.1371/journal.pone.0023476 (PMC3155568; doi:10.1371/journal.pone.0023476)

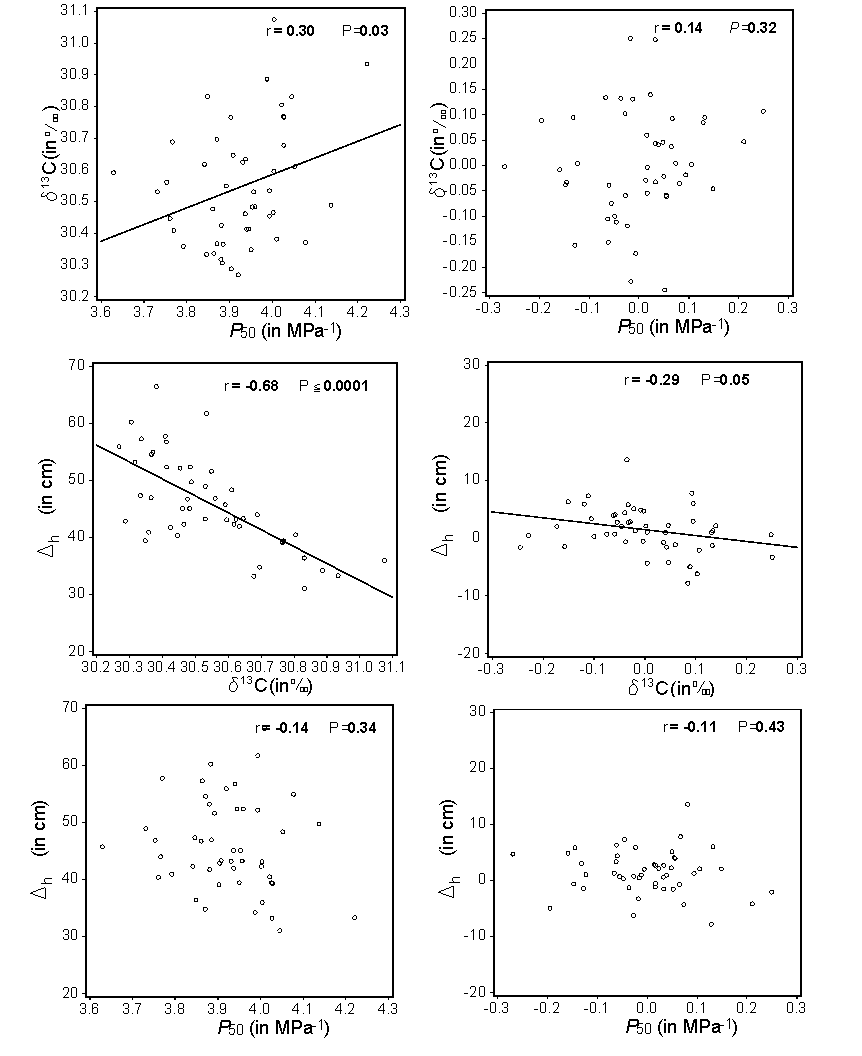

Supplement: Figure S1 — Genetic (right panel) and phenotypic (left panel) correlation between traits. For ease of interpretation, we have converted all the negative values to positive values (P50, δC13). For the genetic correlation, all Pearson correlations (r) were computed over the best linear unbiased prediction (BLUP) (n = 48 for P50, δ13 and n = 151 for Δh). For phenotypic correlation, all Pearson correlations were computed over the BLUP family plus BLUP population and the grand-mean, to ensure that the order of degree of freedom remained the same and the block effects are removed. P50, pressure at 50 % loss of conductivity in MPa, Δh the annual increment between 2004 and 2005, in mm, δ13C is the isotope discrimination for carbon 13 in ‰. (TIF) [file pone.0023476.s001.tif]
