# Supplementary material for: Uniform Selection as a Primary Force Reducing Population Genetic Differentiation of Cavitation Resistance across a Species Range
Source: PLoS One. 2011 Aug 12;6(8):e23476. doi: 10.1371/journal.pone.0023476 (PMC3155568; doi:10.1371/journal.pone.0023476)
Supplement: Table S1 — Review of intraspecific studies for cavitation resistance estimated using P 50 or related parameters (as indicated in the table). Npop: number of populations used, Nind: number of individuals per population used to assess cavitation resistance. The table is divided in two parts, the first part corresponds to provenance or progeny trials, and the second to “in situ” studies. (DOC) [file pone.0023476.s002.doc]

**Table S1: Review of intraspecific studies for cavitation resistance estimated using *P*_50_** or related parameters (as indicated in the table).

| **Provenance trial** | Npop | Nind | Traits | Organ | References |
| --- | --- | --- | --- | --- | --- |
| *Pinus ponderosa* | 4 | 10 | P50 | Stem/root | [1] |
| *Quercus wislizenii* | 3 | 6 | P50 | Stem | [2] |
| *Pinus contorta var latifolia* | 4 | 15 | P50/Slope | Stem | [3] |
| *Pinus pinaster* | 4 | 5 | P50 /Slope | Stem | unpublished |
| *Saccharum sp* | 0 | 4 | P10/P50/P88 | Leaf | [4] |
| *Olea europaea* | 2 | 2 | P50 | Stem | [5] |
| *populus trichocarpa* | 4 | 5 | P12/Slope | Stem | [6] |
| *Ambrosia dumosa* | 3 | 6 | P50 | Stem | [7] |
| *Hymenoclea salsola* | 3 | 6 | P50 | Stem | [7] |
| *Artemisia tridentata* | 3 | 10 | P50 | Stem | [8] |
| **In situ** |  |  |  |  |  |
| *Pinus sylvestris* | 12 | 6.8 | P50 | Branch | [9] |
| *Acer grandidentatum* | 2 | 9 | P50 | Stem/root | [10] |
| *Pinus sylvestris* | 2 | 10 | P50/Slope | Branch | [11] |
| *Pinus ponderosa* | 2 | 6 | P50/Slope | Branch | [12] |
| *Cordia alliodora* | 3 | 3 | P50 | Branch | [13] |
| *Artemisia tridentata* | 3 | 10 | P50 | Stem | [8] |
| *Juniperus scopulorum* | 2 | 12 | P50/Slope | Stem/root | [14] |
| *Fagus sylvatica* | 5 | 25 | P50 | Branch | [15] |

Npop: number of populations used, Nind: number of individuals per population used to assess cavitation resistance. The table is divided in two parts, the first part corresponds to provenance or progeny trials, and the second to “*in situ*” studies.

Table S1 References

1. Kavanagh KL, Bond BJ, Aitken SN, Gartner BL, Knowe S (1999) Shoot and root vulnerability to xylem cavitation in four populations of Douglas-fir seedlings. Tree Physiology 19: 31-37.

2. Matzner SL, Rice KJ, Richards JH (2001) Intra-specific variation in xylem cavitation in interior live oak (*Quercus wislizenii* A. DC.). Journal of Experimental Botany 52: 783-789.

3. Wang JL, Whitlock MC (2003) Estimating effective population size and migration rates from genetic samples over space and time. Genetics 163: 429-446.

4. Neufeld HS, Grantz DA, Meinzer FC, Goldstein G, Crisosto GM, et al. (1992) Genotypic variability in vulnerability of leaf xylem to cavitation in water-stressed and well-irrigated sugarcane. Plant Physiology 100: 1020-1028.

5. Ennajeh M, Tounekti T, Vadel AM, Khemira H, Cochard H (2008) Water relations and drought-induced embolism in olive (*Olea europaea*) varieties 'Meski' and 'Chemlali' during severe drought. Tree Physiology 28: 971-976.

6. Sparks JP, Black RA (1999) Regulation of water loss in populations of *Populus trichocarpa*: the role of stomatal control in preventing xylem cavitation. Tree Physiology 19: 453-459.

7. Mencuccini M, Comstock J (1997) Vulnerability to cavitation in populations of two desert species, *Hymenoclea salsola* and *Ambrosia dumosa*, from different climatic regions. Journal of Experimental Botany 48: 1323-1334.

8. Kolb KJ, Sperry JS (1999) Differences in drought adaptation between subspecies of sagebrush (*Artemisia tridentata*). Ecology 80: 2373-2384.

9. Martinez-Vilalta J, Cochard H, Mencuccini M, Sterck F, Herrero A, et al. (2009) Hydraulic adjustment of Scots pine across Europe. New Phytologist 184: 353-364.

10. Alder NN, Sperry JS, Pockman WT (1996) Root and stem xylem embolism, stomatal conductance, and leaf turgor in *Acer grandidentatum* populations along a soil moisture gradient. Oecologia 105: 293-301.

11. Martinez-Vilalta J, Pinol J (2002) Drought-induced mortality and hydraulic architecture in pine populations of the NE Iberian Peninsula. Forest Ecology and Management 161: 247-256.

12. Maherali H, Williams BL, Paige KN, Delucia EH (2002) Hydraulic differentiation of Ponderosa pine populations along a climate gradient is not associated with ecotypic divergence. Functional Ecology 16: 510-521.

13. Choat B, Sack L, Holbrook NM (2007) Diversity of hydraulic traits in nine Cordia species growing in tropical forests with contrasting precipitation. New Phytologist 175: 686-698.

14. Ogle K, Barber JJ, Willson C, Thompson B (2009) Hierarchical statistical modeling of xylem vulnerability to cavitation. New Phytologist 182: 541-554.

15. Herbette S, Wortemann R, Awad H, Huc R, Cochard H, et al. (2010) Insights into xylem vulnerability to cavitation in *Fagus sylvatica* L.: phenotypic and environmental sources of variability. Tree Physiology.
