# Supplementary material for: Uniform Selection as a Primary Force Reducing Population Genetic Differentiation of Cavitation Resistance across a Species Range
Source: PLoS One. 2011 Aug 12;6(8):e23476. doi: 10.1371/journal.pone.0023476 (PMC3155568; doi:10.1371/journal.pone.0023476)
Supplement: Table S2 — Result of the principal component (PC) analysis (PCA) for climatic data of Pinus pinaster populations (listed in the methods section, n = 763). Contributions to the first, second, third and fourth axes are indicated for each variable (PC1, PC2, PC3, PC4). The eigenvalues of PC1 = 7.65, PC2 = 3.059, PC3 = 0.97, PC4 = 0.86. W is mean wet ground days (days). I is mean Martonne's index (Pi/(Ta+10)). Pi is the mean precipitation (mm.days−1). C is percent of cloud cover (%). S is the mean of wind speed (m.s−1). V is the water vapor pressure in air (hPa). VPD is the water vapor pressure deficit of air (hPa). T min is the minimum temperature (°C). ΔDT is the mean diurnal temperature range (°C). T m is mean temperature (°C). RG is mean global radiation (W.m2). T max is the maximum temperature (°C). H is mean soil water deficit (Pi-ETP, in mm). ETP is mean Truc's potential evapotranspiration (mm). (DOC) [file pone.0023476.s003.doc]

**Table S2. Result of the principal component (PC) analysis (PCA) for climatic data of *Pinus pinaster* populations (listed in the methods section, n=763).**

| Annual average | *W* | *I* | *P*_i_ | *C* | *S* | *V* | *VPD* | *T*_min_ | *∆*_DT_ | *T*_m_ | *R_G_* | *T*_max_ | *H* | *ETP* |
| --- | --- | --- | --- | --- | --- | --- | --- | --- | --- | --- | --- | --- | --- | --- |
| PC1 | -0.33 | -0.32 | -0.29 | -0.29 | -0.17 | 0.06 | 0.10 | 0.18 | 0.22 | 0.27 | 0.31 | 0.31 | 0.34 | 0.34 |
| PC2 | 0.07 | 0.10 | 0.23 | 0.24 | -0.05 | 0.54 | -0.04 | 0.49 | -0.31 | 0.37 | -0.21 | 0.22 | -0.03 | 0.12 |
| PC3 | 0.06 | 0.10 | 0.08 | 0.02 | 0.48 | 0.04 | 0.86 | 0.01 | 0.04 | 0.02 | 0.03 | 0.03 | 0.09 | 0.03 |
| PC4 | -0.14 | 0.17 | 0.15 | -0.25 | 0.71 | -0.03 | -0.44 | 0.09 | -0.18 | 0.01 | 0.28 | -0.05 | 0.14 | 0.16 |

Contributions to the first, second, third and fourth axes are indicated for each variable (PC1, PC2, PC3, PC4). The eigenvalues of PC1 = 7.65, PC2 = 3.059, PC3 = 0.97, PC4 = 0.86. *W* is mean wet ground days (days). *I* is mean Martonne’s index (P_i_/(T_a_+10)). *P_i_* is the mean precipitation (mm.days^-1^). *C* is percent of cloud cover (%). *S* is the mean of wind speed (m.s^-1^). *V* is the water vapor pressure in air (hPa). *VPD* is the water vapor pressure deficit of air (hPa). *T*_min_ is the minimum temperature (°C). *∆*_DT_ is the mean diurnal temperature range (°C). *T*_m_ is mean temperature (°C). *R_G_* is mean global radiation (W.m^2^). *T*_max_ is the maximum temperature (°C). *H* is mean soil water deficit (P_i_-ETP, in mm). *ETP* is mean Truc’s potential evapotranspiration (mm).
